# Response of Species Diversity and Stability of Typical Steppe Plant Communities on the Mongolian Plateau to Different Grazing Patterns

**DOI:** 10.1002/ece3.70360

**Published:** 2024-09-30

**Authors:** Suriguga Gao, Yu Hong, Wulan Tuya, Weiqing Zhang, Chang An, Siqin Chaoketu, Xu Sha, Bu He, Wu Yinga

**Affiliations:** ^1^ College of Geographic Sciences Inner Mongolia Normal University Hohhot China; ^2^ Key Laboratory of Mongolian Plateau Disaster and Ecological Safety Hohhot China; ^3^ Key Laboratory of Climate Change and Regional Response on the Mongolian Plateau Hohhot China; ^4^ College of Life Science and Technology Inner Mongolia Normal University Hohhot China

**Keywords:** community stability, community structure, different grazing patterns, species diversity, typical steppe of the Mongolian plateau

## Abstract

Grasslands represent a major biome on Earth and play a vital role in ecosystem functioning and dynamics. However, owing to the variations among grassland types, the impact of grazing on plant community diversity and stability remains unclear. This study is based on the typical steppe of the Mongolian Plateau. Field sampling and data analysis were combined to qualitatively and quantitatively investigate the structural characteristics, species diversity, and stability of plant communities under varying grazing intensities, that is, four‐season nomadic, two‐season rotational, and sedentary grazing (FSNG, TSRG, and SG, respectively). The results indicated that FSNG pastures exhibited the largest number of plant species while FSNG and TSRG pastures exhibited relatively high importance values for the primary dominant species. *Carex duriuscula*, *Chenopodium glaucum*, and *Cleistogenes squarrosa* were prominent in SG pastures, with *C. duriuscula* having the largest importance value. The mean height, cover, and aboveground biomass of plant communities in FSNG were significantly higher than those in SG (*p* < 0.05), with no significant difference observed between FSNG and TSRG. FSNG also demonstrated the highest Shannon–Wiener, Simpson, and Pielou indexes. The Shannon–Wiener and Simpson indexes between the FSNG, TSRG, and SG pastures showed significant differences (*p* < 0.05). Nomadic plant communities displayed positive loosely interspecific traits, suggesting independence and positive succession. Conversely, communities in TSRG and SG exhibited negative correlations and higher instability. The stability analysis ranked community stability as FSNG > TSRG > SG, suggesting that judicious grazing practices could enhance grassland stability. The findings reveal that grazing patterns influence plant community composition and function and that FSNG pastures promote higher species diversity, perennial dominance, and overall stability compared with TSRG and SG pastures.

## Graphical abstract

This paper focuses on the Wuzhumuqin–Wendurhan Sample Strip, a typical grassland on the Mongolian Plateau, and explores the structural characteristics, species diversity, and stability of plant communities in response to different grazing practices, which is relevant to the scope of the journal's theme.

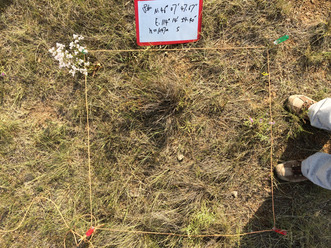

## 1 Introduction

Plant community diversity is a vital aspect of biodiversity and plays a key role in supplying material and energy to ecosystems, maintaining ecosystem stability, and regulating global climate (Zhao et al. 2023). Community stability encompasses the system's capacity to withstand external disturbances and includes factors such as resilience, resistance, and persistence (Yao et al. 2016; Ishii et al. 2021). Grazing exerts a widespread influence on the Mongolian Plateau, impacting not only species composition, community diversity, and ecosystem stability but also material cycles and energy flows. Consequently, it stands as a pressing concern within the international ecological community (Briske et al. 2008; Conte and Tilt 2014; Zhang et al. 2022). In recent years, there has been a surge in research focusing on precise grazing management systems. Controlled experiments have scrutinized nomadic, rotational, and continuous grazing to ascertain their respective advantages and disadvantages (Jacobo et al. 2006; Na, Bao, et al. 2018). Despite these efforts, the effects of varied grazing practices on community diversity and stability within typical Mongolian Plateau grasslands remain inadequately elucidated (De Boeck et al. 2018; Valerio et al. 2022).

The Mongolian Plateau is located in Central Asia and has a typical continental arid and semiarid climate that is sensitive to climate change and exhibits fragile ecosystems (Zhang et al. 2009). Typical grasslands of the Mongolian Plateau are present in Sukhbaatar, Kent, and Dongfang Provinces, as well as in the eastern part of the Xilin Gol League and western part of Hulunbeier City in Inner Mongolia, and represent the core components of the temperate grasslands (Wulan 2021). The Inner Mongolian Steppe represents the southern part of this steppe belt, serving as one of China's major animal husbandry bases and an important ecological barrier in northern China (Wulan 2021). However, owing to the wide distribution area of grasslands and the intensity of their utilization, the issues of grassland desertification and vegetation degradation are becoming increasingly serious (Li and Qu 2002; Liu et al. 2009; Zhang et al. 2018). Such changes are threatening the ecological security, social stability, and economic development of China's northern border (Bao, Liu, et al. 2013; Zhang et al. 2018).

Grazing is the primary method of grassland utilization on the Mongolian Plateau, constituting a significant ecological disturbance. This disturbance can profoundly affect entire ecosystems by modifying soil physicochemical properties and increasing habitat heterogeneity, thereby influencing the distribution of species as well as the structure and diversity patterns of grassland communities. Notably, the pasture contracting system introduced in Inner Mongolia during the early 1980s has led to a gradual transition in grassland utilization from nomadic pastoralism to sedentary pastoralism (Na, Li, et al. 2018). This shift encompasses diverse approaches, such as settled grazing, restricted grazing, fallow pastoralism, and rotational pastoralism, thereby deviating from the traditional nomadic pastoralism practiced in Mongolia. This change exposed the grassland zone to different pressures and influencing factors, which decreased grassland productivity, escalated grass–animal conflict, and reduced vegetation diversity and stability (Liu et al. 2009; Na, Li, et al. 2018), posing a serious threat to the sustainable development of grassland animal husbandry. Therefore, in recent years, Mongolian Plateau vegetation has become a focus of many researchers, both at home and abroad, who have studied the impact of grazing on plant community diversity and stability. Studies have suggested that short‐term grazing exclusion, rotational grazing, deferred grazing, and moderate disturbance levels are beneficial for maintaining species diversity (Zhao et al. 2023). However, other research indicates that long‐term grazing exclusion can lead to an excessive increase in the dominance of one or a few species, thereby reducing grassland species richness and diversity (Wang et al. 2019). Additionally, continuous grazing degrades grassland habitats, thus causing significant shifts in the dominance of key functional plant groups and reducing community species diversity and structural complexity. Grazing disturbance is one of the factors that influences grassland ecosystem stability, and it has a direct impact on community stability (Zhao et al. 2023). Studies have shown that as grazing intensity increases, community functional diversity and stability significantly decrease (Shen, Wang, and Han 2022). Under varying degrees of grazing disturbance, the stability of desert grassland ecosystems differs, with excessive grazing pressure negatively affecting grassland productivity and ecological functions. However, due to limitations imposed by international borders, communication barriers, and adverse weather conditions, research is often confined to small areas within a single country (Yin et al. 2013). Changes in typical steppe ecosystems of the Mongolian Plateau are primarily studied using remote sensing images to extract the normalized difference vegetation index (NDVI) of grassland vegetation and analyze spatial and temporal variations (Bao, Bao, et al. 2013; Batunacun et al. 2015; Tao et al. 2015). However, comprehensive research on the effects of different grazing practices on species diversity and ecosystem stability across the transboundary typical steppe of China and Mongolia is still lacking.

Plant community structure, species diversity, and stability constitute fundamental components of community ecology, thereby serving as crucial indicators for assessing and managing degraded grassland resources (Miao et al. 2014). Therefore, studying the community structure and stability is important for understanding and protecting grassland ecosystems, and further investigations are warranted on the effects of grazing methods on plant community species diversity and stability in typical grasslands on the Mongolian Plateau.

This study aimed to determine whether unwarranted grazing practices (1) degrade the structural characteristics of the plant community in the direction of succession, (2) reduce the species diversity of plant communities, or (3) decrease plant community stability. Determining the most sensible grazing practices will help achieve the sustainable use of typical grasslands on the Mongolian Plateau. Furthermore, our findings will provide a reference for the restoration and stabilization of grassland ecosystems.

## 2 Materials and Methods

### 2.1 Sample Zone Delineation

**FIGURE 1:**
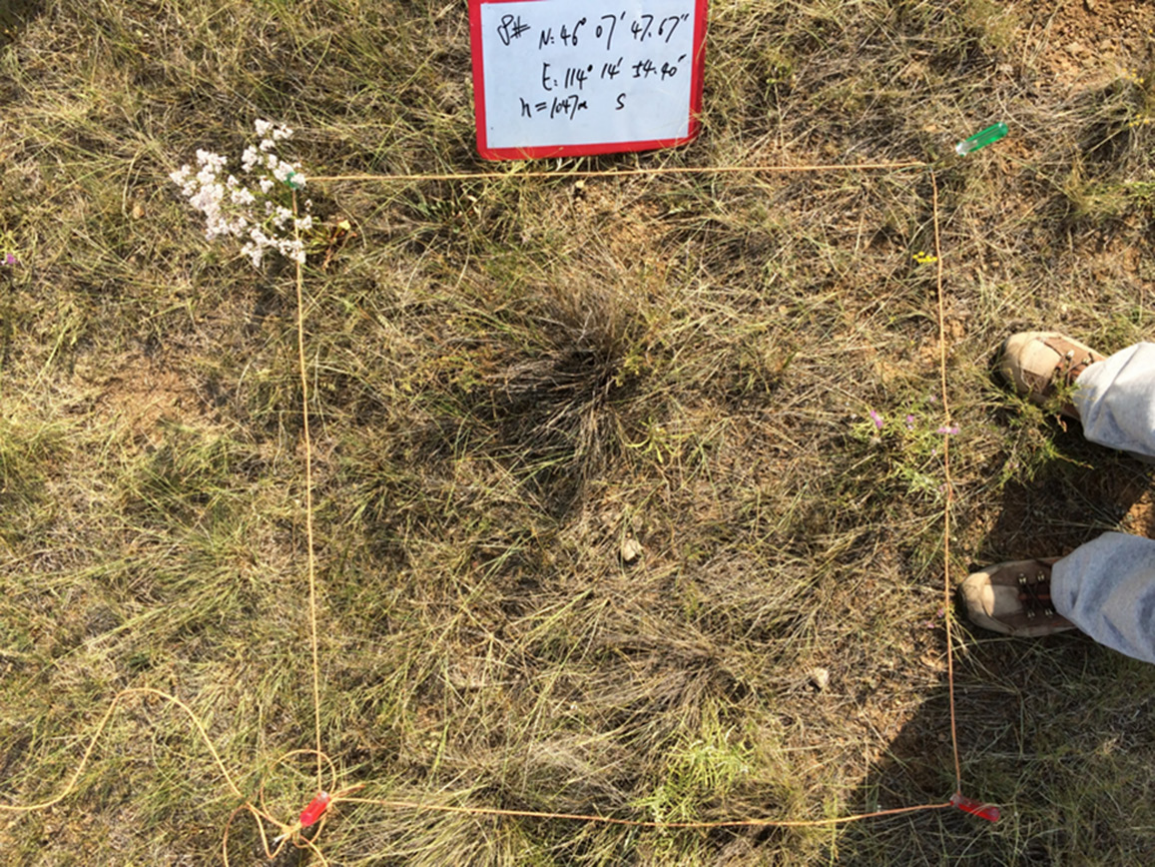
Setup of plant sample plots at the sampling site.

**FIGURE 2:**
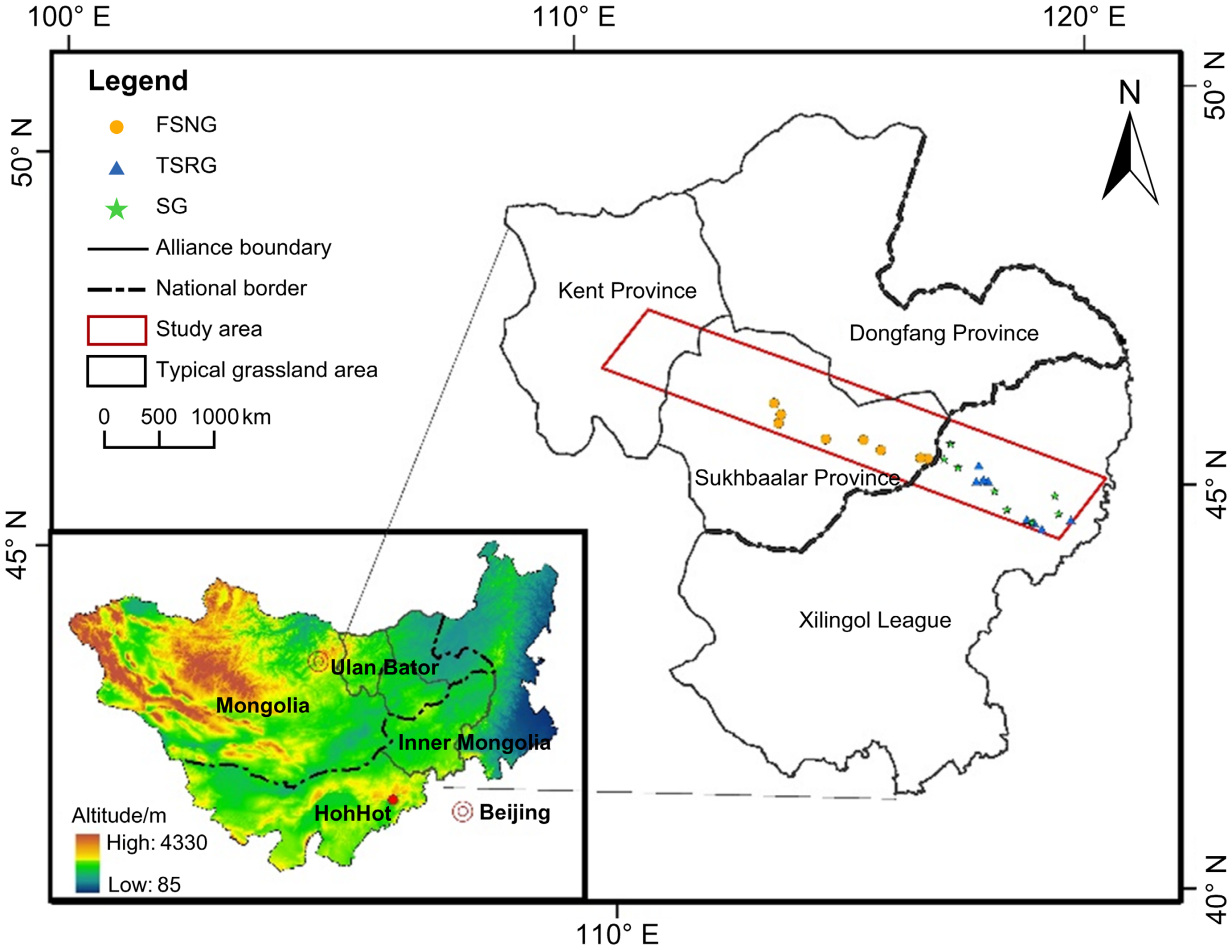
Study area location. FSNG, four‐season nomadic grazing; SG, settled grazing; TSRG, two‐season rotational grazing.

The study describes the setup of plant sample plots and the recording of geographic information at the sampling site (Figure 1). The vegetation type within the sample plot is herbaceous, with specific geographic coordinates and heights noted on a whiteboard. Based on the unified context of a typical steppe–chestnut–calcium soil operating area, the delineation of sample zones prioritized the homogeneity of subsurface natural geographic conditions, particularly focusing on selecting high plains with flat topography. Additionally, the sample zones were strategically positioned to span across territories of different countries, ensuring a comprehensive representation of the changing characteristics of the same grassland under varied country borders, utilization modes, and intensities (Wulan 2021). Specifically, the sample zone encompasses the core area of the typical steppe on the Mongolian Plateau, stretching from the city of Wendurhan, Kent Province, in northwestern Mongolia, to Bayanhua Town, Xiwuzhumuqin Banner, in Inner Mongolia, China. The sample encompasses four control points: (111°8′51.25″ E, 47°56′20.13″ N) (northwest), (119°13′25.31″ E, 45°13′9.35″ N) (northeast), (118°14′32.64″ E, 44°32′30.80″ N) (southeast), (110°13′34.08″ E, 47°13′58.02″ N) (southwest). These points span diagonally across the Kent, Sukhbaatar, and Dongfang Provinces of Mongolia and the Xilinguole League of Inner Mongolia, with a total area of 69,850,000 km^2^ (Figure 2). Within the sample zone, 66.73% of the area is in Mongolia, and 33.27% is in Inner Mongolia (Wulan 2021).

### 2.2 Experimental Design and Field Sampling

While the sample plots in this study are situated in various regions of China and Mongolia, they share similar topographies, soil compositions, plant diversity, grazing levels, and livestock demographics. To mitigate the impact of environmental variables, this paper performed a significance analysis on the ecological factors of the study area samples, as shown in Table 1. Interviews with herders and field surveys revealed that three types of grazing practices are common in the study area: four‐season nomadic grazing (FSNG), two‐season rotational grazing (TSRG), and settled grazing (SG). In FSNG areas, herders migrate according to seasonal changes and typically utilize four pastures for spring, summer, autumn, and winter. In TSRG areas, herders alternate pastures between the spring and autumn seasons or between the summer and winter seasons. In SG areas, herders grazing in a fixed location, with the pasture position remaining largely unchanged.

**TABLE 1:** Basic information of the sample plots.

Sample plot	Altitude (m)	Average stocking rate (sheep unit/km^2^)	Annual mean temperature (°C)	Annual total precipitation (mm)	Soil type	Sample size	Main dominant plants
FSNG	1020a	0.14a	1.92a	244.41a	Castanoems	8	*Leymus chinensis*, *Stipa grandis*, *Cleistogenes squarrosa*
TSRG	939.5a	0.16a	1.90a	234a	Castanoems	8	*C. squarrosa*, *L. chinensis*, *Chenopodium glaucum*
SG	962.2a	0.15a	1.90a	234a	Castanoems	8	*Carex duriuscula*, *C. squarrosa*, *C. glaucum*

*Note:* Lowercase letters indicate significant differences at the 5% level. *Source:* Meteorological Bureau of Xilingol League and National Agency for Meteorology and Environmental Monitoring of Mongolia. Stocking rate—total number of livestock (sheep) owned by herders in the sample plot/total area of grassland owned by herders. All types of livestock were converted to sheep units according to China's sheep unit conversion standard as follows: one camel—seven sheep, one horse—six sheep, one cow—five sheep, and one goat—one sheep.

During July and August of 2016 and 2017, we surveyed plant communities in the study area, specifically in the herdsmen's pastures, and established a total of 24 sampling points, with eight points randomly selected within each of the three grazing types. At each sampling point, which covered a 1 m^2^ plot. In sedentary and two‐season rotational grazing systems, the distances between adjacent sampling points were generally consistent, typically within 200 m. In contrast, the distance between adjacent sampling points was increased to effectively capture the spatial variability present within these pastures owing to the high heterogeneity of the four‐season nomadic pastures. In these plots, we assessed various parameters, including plant height, density, cover, aboveground biomass, and species composition. Plant height was measured from the base to the canopy using a tape, with average heights calculated from 3 to 5 specimens per species. Plant density was determined by counting the total number of plants within the sample area. To estimate vegetation cover, we captured photographs of the samples from a perpendicular angle and used the Photoshop 6 software to calculate the pixels occupied by plants based on the color contrast between plants and soil. The ratio of plant pixels to the total pixels of the sample provided the cover value. Aboveground biomass was obtained using a harvesting method for each sample plot. The fresh weight of each plant by species was measured, and samples were transported to the laboratory. The plants were subsequently dried to a constant weight at 65°C, and their dry weight was measured (Jian et al. 2018).

### 2.3 Data Processing and Methods

#### 2.3.1 Community Species Importance Values and Plant Diversity

In this study, species occurring in the plant community were categorized into four functional groups according to differences in life type: perennial grasses, perennial forbs, annual and biennial plants, shrubs, and semishrubs (Jian et al. 2018; Sasaki et al. 2019; Zhang et al. 2020; Suriguga et al. 2022). Diversity indexes are typically used to represent plant community diversity (Sun, Zhu, and Zhang 2014). Thus, the Shannon–Wiener, Margalef, Pielou, and Simpson indexes and the dominance of species in a plant community were determined based on the importance value (p_i_) (Jian et al. 2018).

The associated formulae are provided below.
(1)

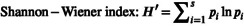

(2) $$\text{Margalef index}:\mathrm{MA}=\left(S-1\right)/\ln\;N$$

(3) Pielou index:E=H/lnS

(4) $$\text{Simpson index}:p=1-\sum_{i=1}^{s}p_{i}^{2}$$

where *S* denotes the number of species in the plant community, *N* denotes the sum of the number of individuals of all species, and *p*_*i*_ denotes the species importance value.

Next, we measured the β‐diversity indexes of plant communities among different grazing patterns in typical grasslands in China and Mongolia. A simpler measure of plant community similarity coefficients was used, that is, the Jaccard coefficient (Jian et al. 2018).

(5) $$\text{Jaccard coefficient}:\beta_{j}=\frac{c}{a+b-c}$$

where *a* and *b* are the number of species in the two plant communities in different habitats, and c is the number of species common to the two plant communities. Additionally, *β* ranges from (0, 1), where *β* values in the range of (0, 0.25) indicate that the two communities are extremely dissimilar; (0.25, 0.50), the two communities are moderately dissimilar; (0.5, 0.75), the two communities are moderately similar; and (0.75, 1.00), the two communities are extremely similar (Bátori et al. 2012).

#### 2.3.2 Overall Community Correlation Analysis

The correlation among plant species illustrates their adaptation to various grazing patterns in China and Mongolia's typical grasslands, effectively depicting plant community structures. In this study, the variance ratio method derived from the null‐linkage model (Roxburgh, Shea, and Wilson 2004) was used to quantitatively verify the overall correlation of typical steppe plant communities in China and Mongolia.

(6) $$\text{Variance calculations}:\delta_{T}^{2}=\sum_{i=1}^{s}p_{i}\left(1-p_{i}\right),S_{T}^{2}=\left(\frac{1}{N}\right)\sum_{j-1}^{N}{\left(T_{j}-t\right)}^{2}$$

(7) $$\text{Variance ratio}:\mathrm{VR}=\frac{S_{T}^{2}}{\delta_{T}^{2}}$$

where *S* is the total number of species, *p*_*i*_ = *n*_*i*_/*N*, *n*_*i*_ is the number of samples where species *i* occurs, and *N* is the total number of samples; *T*_*j*_ is the total number of species occurring in sample plot *j*; *t* is the average number of species within the sample; VR is the overall association index between species within the community; VR > 1 indicates that the species shows an overall positive association, VR = 1 indicates no association between all species, and VR < 1 indicates an overall negative association between species; and *W* is used to test the significance of the VR offset 1, *W* = VR × *N*. If the interspecific association is not significant, there is a 90% probability that *W* falls into *χ* ^2^0.05(*N*) < *W* < *χ* ^2^0.95(*N*) (Jian et al. 2018).

#### 2.3.3 Community Stability Analysis

We employed the Godron contribution law method to rank plant species surveyed under different grazing practices by frequency and convert them to relative frequency for stepwise accumulation. This involved calculating the cumulative sum of the plant species in their sorted order and expressing it as percentages. Subsequently, we generated a fuzzy scatter smoothing curve, plotting the cumulative inverse percentage of plant species on the *x*‐axis against the cumulative relative frequency on the *y*‐axis. This visualization method effectively illustrates the relationship between grazing practices and the distribution of plant species across the study area. Finally, coordinates of the intersection point with the *x* + *y* = 100 line were found, which represent the stabilization point of the desired plant community. The closer the cumulative inverse percentage of plant species and the cumulative relative frequency percentage of the plant community is to 20/80, the more stable the plant community will be (Huang et al. 2015; Linders et al. 2019).

(8) $$\text{Smooth curve and linear models}:y={\mathrm{ax}}^{2}+\mathrm{bx}+c,y=100-x$$

(9) $$\text{Solution formula}:{\mathrm{ax}}^{2}-\left(b+1\right)x+100-c=0$$

The coordinates of the intersection point (*x*, *y*) were derived from Equation (9) and compared with 20/80 to determine the degree of stabilization of the plant community for different grazing practices.

Excel (2007) and SPSS (22.0) were used for data processing and statistical analyses, respectively. The *t*‐test was used for identifying significant differences between the two variables (*α* = 0.05), and Origin 9.1 software was used for plotting.

## 3 Results

### 3.1 Changes in Plant Community Characteristics Under Different Grazing Methods

#### 3.1.1 Plant Community Composition and Structure

Grassland plant community composition determines the community structure, appearance, function, and succession direction and thus is one of the most intuitive characteristics of vegetation (Gao et al. 2019; Chang et al. 2020). The choice of grazing method influences the composition of plant communities, as evidenced by the total numbers of species observed in the FSNG, TSRG, and SG areas, at 37, 27, and 23, respectively (Table 2). In terms of species importance value, dominant species differed across the grazing areas. In the FSNG area, *Leymus chinensis*, *Stipa grandis*, and *Carex duriuscula* held the highest importance value at 49.16%. Conversely, the TSRG area was characterized by brown *Cleistogenes squarrosa*, *L. chinensis*, and *Chenopodium glaucum*, with an importance value of 35.19%. Finally, the SG area was dominated by *C. duriuscula*, *C. squarrosa*, and *C. glaucum*, with an importance value of 47.73%. The results reveal differences among the dominant species of typical steppe of FSNG, TSRG, and SG. The dominant species in FSNG pastures remained relatively stable, whereas, in TSRG, *L. chinensis* and *C. squarrosa* dominated, with a shift in the relative importance of the species. However, the presence of degradation indicator plants such as *C. duriuscula* and *C. glaucum* in the SG area indicated severe degradation.

**TABLE 2:** Composition and importance values of typical steppe communities in China and Mongolia.

Functional group	Species	Importance value %		
		FSNG	TSRG	SG
Perennial grasses	*Leymus chinensis*	19.97	11.28	10.66
	*Stipa grandis*	13.85	3.45	8.63
	*Cleistogenes squarrosa*	11.59	14.22	15.43
	*Stipa breviflora*	2.34	—	—
	*Stipa mongolorum* Tzvel.	4.64	—	—
	*Agropyron cristatum*	1.45	1.09	1.93
	*Achnatherum splendens*	1.71	—	—
	*Stipa krylovii*	—	9.58	9.53
Perennial forbs	*Stellera chamaejasme* Linn	—	3.58	—
	*Allium tenuissimum*	1.19	2.03	2.29
	*Allium ramosum*	—	1.45	—
	*Iris tenuifolia* Pall.	—	—	1.00
	*Carex duriuscula*	15.34	9.36	18.07
	*Allium polyrhizum*	3.45	6.91	1.33
	*Saussurea mongolica*	1.08	—	—
	*Asparagus dauricus* Link	0.33	—	1.21
	*Convolvulus ammannii*	1.12	1.69	1.62
	*Serratula komarovii*	1.22	1.96	1.43
	*Anemarrhena asphodeloides* Bunge	—	3.91	—
	*Allium ramosum*	—	—	2.11
	*Bromus inermis* Leyss.	—	—	—
	*Thalictrum petaloideum* L.	1.18	—	—
	*Potentilla acaulis*	—	—	1.1
	*Astragalus melilotoides* Pall	—	1.05	1.83
	*Allium mongolicum* Regel	1.06	—	—
	*Gymnocarpos przewalskii*	3.53	—	—
Shrubs and semi‐shrubs	*Caragana microphylla* Lam	3.76	—	—
	*Artemisia frigida*	1.64	4.05	2.96
	*Artemisia halodendron*	—	2.88	1.83
	*Artemisia adamsii* Bess.	—	3.38	—
	*Ptilotricum canescens*	—	2.17	1.35
Annual and biennial plants	*Artemisia annua*	5.67	—	—
	*Chenopodium glaucum*	—	9.69	14.23
	*Artemisia palustris* Linn.	—	—	—
	*Salsola collina*	—	3.38	1.32
	*Lappula myosotis* Moench	—	0.59	2.17
	*Atriplex tatarica* (L.) Bung	1.19	—	1.20

*Note:* “—” indicates a plant community where the species does not occur or has an importance value of 1.

Abbreviations: FSNG, four‐season nomadic grazing; SG, settled grazing; TSRG, two‐season rotational grazing.

**FIGURE 3:**
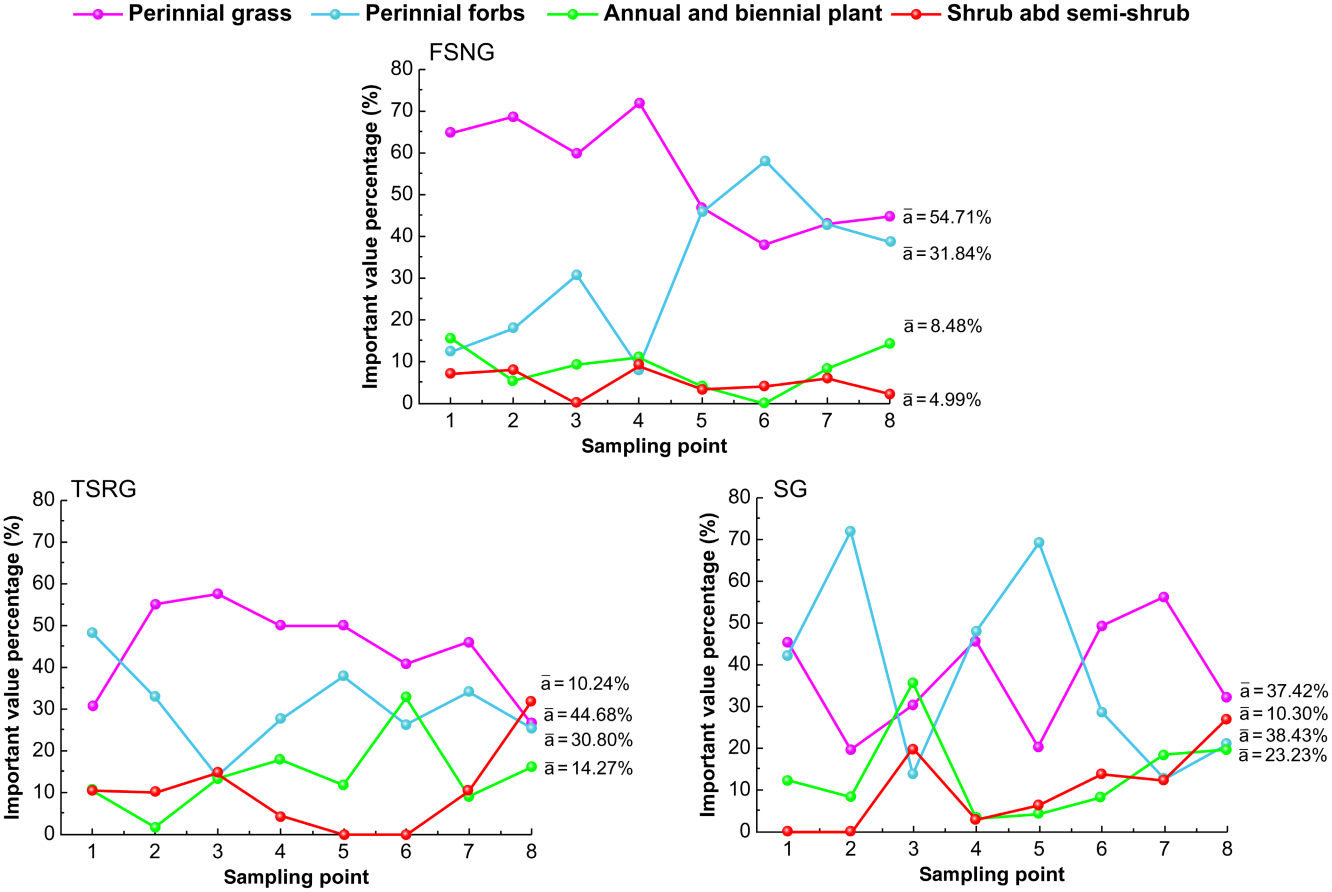
Quantitative characteristics of plant communities in each sample area.

In terms of functional groups of species, FSNG, TSRG, and SG pastures were dominated by perennial grasses and perennial forbs (Figure 3). Among them, the average percentage of perennial grasses in FSNG, TSRG, and SG areas were 54.7%, 44.68%, and 38.43%, respectively.

The mean perennial forbs ratio was 31.84%, 30.80%, and 37.42%, respectively. Shrub and semishrub plants showed importance values of 4.99%, 10.24%, and 10.30%, respectively. The mean values of the proportion of annual and biennial plants were 8.48%, 14.27%, and 23.23%. Furthermore, the order of taxa proportion of the functional groups of grassland communities with different grazing modes was perennial grasses > perennial forbs > annual and biennial plant > shrub and semishrub. However, there was a difference in the mean value of the percentage. Moreover, the structure of sedentary grazing plant communities progressively degraded.

#### 3.1.2 Quantitative Characteristics of the Plant Communities

**FIGURE 4:**
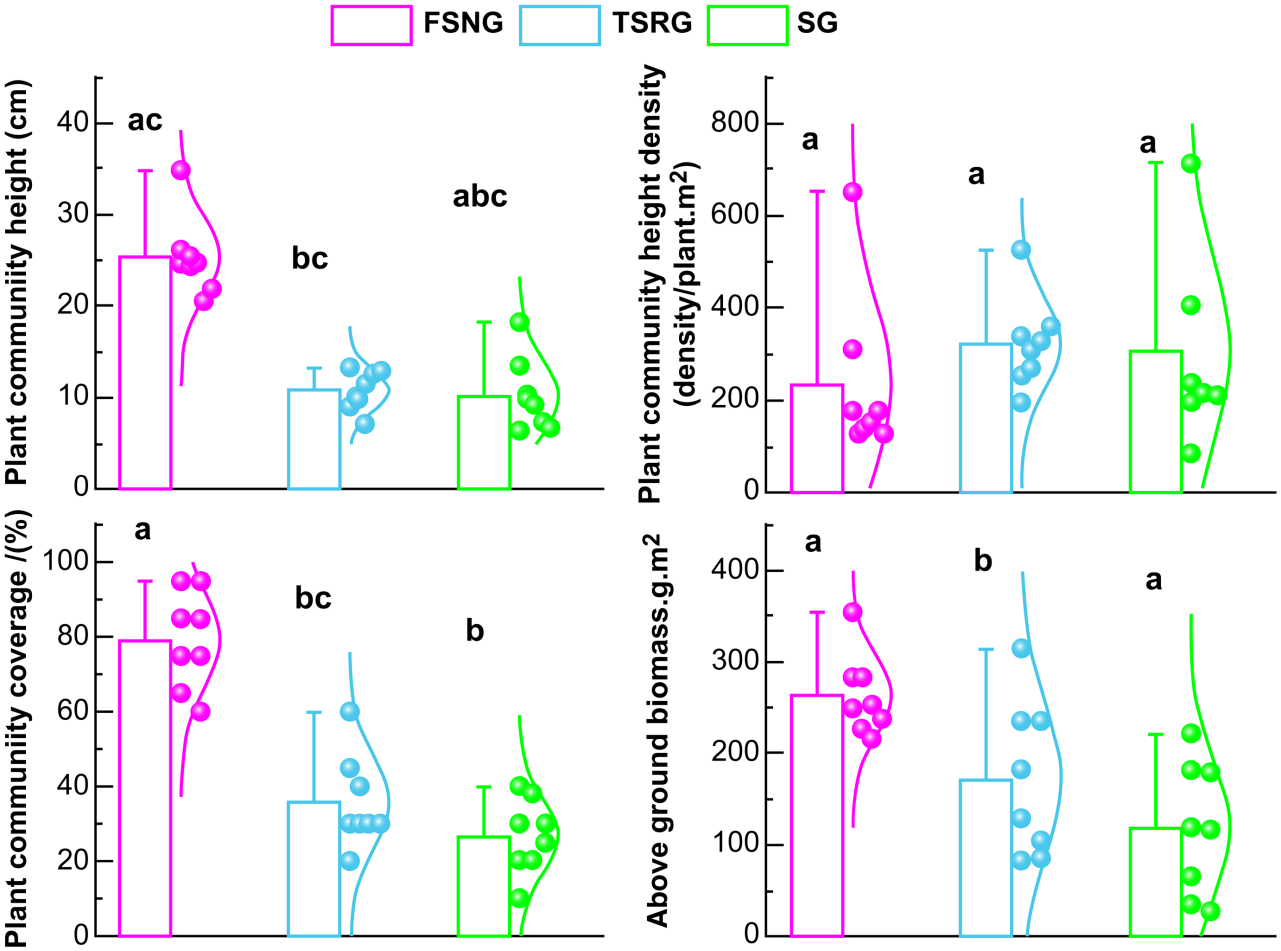
Comparison of the quantitative characteristics of typical steppe communities in China and Mongolia. The small cubes in each box represent mean values. Different lowercase letters indicate significant differences between grazing practices (*p* < 0.05). FSNG, four‐season nomadic grazing; SG, settled grazing; TSRG, two‐season rotational grazing.

Community characterization is the basis for ecological restoration and reestablishment and the integration of vegetation adaptations to ecosystems (Chang et al. 2020). Different grazing methods affect the quantitative characteristics of plant communities (Cumming et al. 2016). The average heights of the FSNG, TSRG, and SG plant communities were 25.30, 10.80, and 10.16 cm, respectively, with a significant difference between FSNG, TSRG, and SG (*p* < 0.05) (Figure 4). This result indicates that the heights of FSNG were higher than that of the other grazing modes and that the heights of the communities were significantly lower, especially in the case of SG conditions (*p* < 0.05). The plant community densities of FSNG, TSRG, and SG were recorded as 228, 317.36, and 303 plants/m^2^, respectively, with no significant difference observed among the three grazing methods (Figure 4). Despite this, TSRG demonstrated the highest density, suggesting its density surpassed that of the other grazing methods. Additionally, the plant community cover for FSNG, TSRG, and SG was reported as 79.37%, 35.62%, and 28.11%, respectively, with significant differences noted between the FSNG community and TSRG and SG communities, while no significant differences were observed between TSRG and SG communities (*p* < 0.05). This outcome implies that the community cover of the FSNG plot exceeded that of the TSRG and SG plots. The aboveground biomass of plant communities in Mongolia was 262.87, 170.58, and 131.74 for FSNG, TSRG, and SG, respectively, and with a significant difference between FSNG, TSRG, and SG (*p* < 0.05) (Figure 3). This result indicates that the aboveground biomass of the FSNG pasture was higher than that of the TSRG and SG Mongolian pastures.

### 3.2 Characterization of Plant Community Diversity

The Shannon–Wiener, Margalef, Pielou, and Simpson community diversity indices were used to characterize the changes in community diversity (Bao et al. 2009; Liu et al. 2022). The Shannon–Wiener, Simpson, and Pielou indexes were the highest for TSRG at 2.14, 0.90, and 0.85, respectively, and the Margalef index was the highest for FSNG at 1.72 (Table 3). The Margalef index was not significantly different across grazing practices; the Shannon–Wiener, Simpson, and Pielou indexes were significantly higher (*p* < 0.05) for FSNG than for TSRG and SG.

**TABLE 3:** Species diversity in typical steppe communities in China and Mongolia.

Test sample	Shannon–Wiener index	Simpson index	Pielou index	Margalef index
FSNG	2.14 ± 0.06a	0.90 ± 0.11a	0.85 ± 0.11a	1.06 ± 0.21a
TSRG	1.95 ± 0.09a	0.83 ± 0.03b	0.83 ± 0.14a	1.72 ± 0.12a
SG	1.70 ± 0.08b	0.87 ± 0.21ab	0.78 ± 0.02ab	1.13 ± 0.10a

*Note:* Lowercase letters indicate significant differences between grazing practices (*p* < 0.05).

Abbreviations: FSNG, four‐season nomadic grazing; SG, settled grazing; TSRG, two‐season rotational grazing.

Jaccard's similarity coefficient was used as a measure of the similarity of typical steppe communities with different grazing practices, and the similarity coefficients were analyzed (Jian et al. 2018). The Jaccard coefficients of FSNG and TSRG pastures were 0.25, indicating moderate dissimilarity; those of FSNG and sedentary grazing were 0.23 (ranging from 0.01 to 0.25), indicating that the communities were at a very dissimilar level, whereas those of TSRG and SG were 0.61 (ranging from 0.5 to 0.75), indicating that the communities were at a moderately similar level (Table 4). Therefore, large habitat variability was observed between the plant communities under different grazing patterns in typical grasslands in China and Mongolia. This result suggests that different grazing practices affected population dispersal and settlement, which in turn changed the composition of plant communities and formed different community structures.

**TABLE 4:** Similarity coefficients of typical steppe communities in China and Mongolia.

Test sample	FSNG	TSRG	SG
FSNG	1	0.25	0.23
TSRG	0.25	1	0.61
SG	0.23	0.61	1

Abbreviations: FSNG, four‐season nomadic grazing; SG, settled grazing; TSRG, two‐season rotational grazing.

### 3.3 Stability Analysis of Plant Community Stability

Under different grazing modes, the plant communities in the FSNG pastures exhibited a VR > 1, indicating that the community populations were generally positively correlated (Table 5). The test value was *W* = 9.52, within the interval of (X20.95, X20.05), indicating that it did not reach the significance level. The community plant populations were in the unstable stage of overall positive succession. TSRG and SG pastures had VR < 1, indicating that the community had overall negative associations. The test values *W* = 5.46 and *W* = 6.42, respectively, fell within the (X20.95, X20.05) interval, indicating that they did not reach the significance level. This result suggests that the community is in the unstable stage of negative succession. Compared with the FSNG areas, the TSRG and SG areas were characterized by looser interspecific linkages, greater independence between species, and plant community degradation risk.

**TABLE 5:** Overall correlation between typical steppe plant communities in China and Mongolia.

Test sample	Variance ratio	Test statistic	X^2^ threshold	Test results
FSNG	1.19	9.52	(2.73, 15.51)	Positive correlation
TSRG	0.683	5.46		Negative correlation
SG	0.802	6.42		Negative correlation

Abbreviations: FSNG, four‐season nomadic grazing; SG, settled grazing; TSRG, two‐season rotational grazing.

**FIGURE 5:**
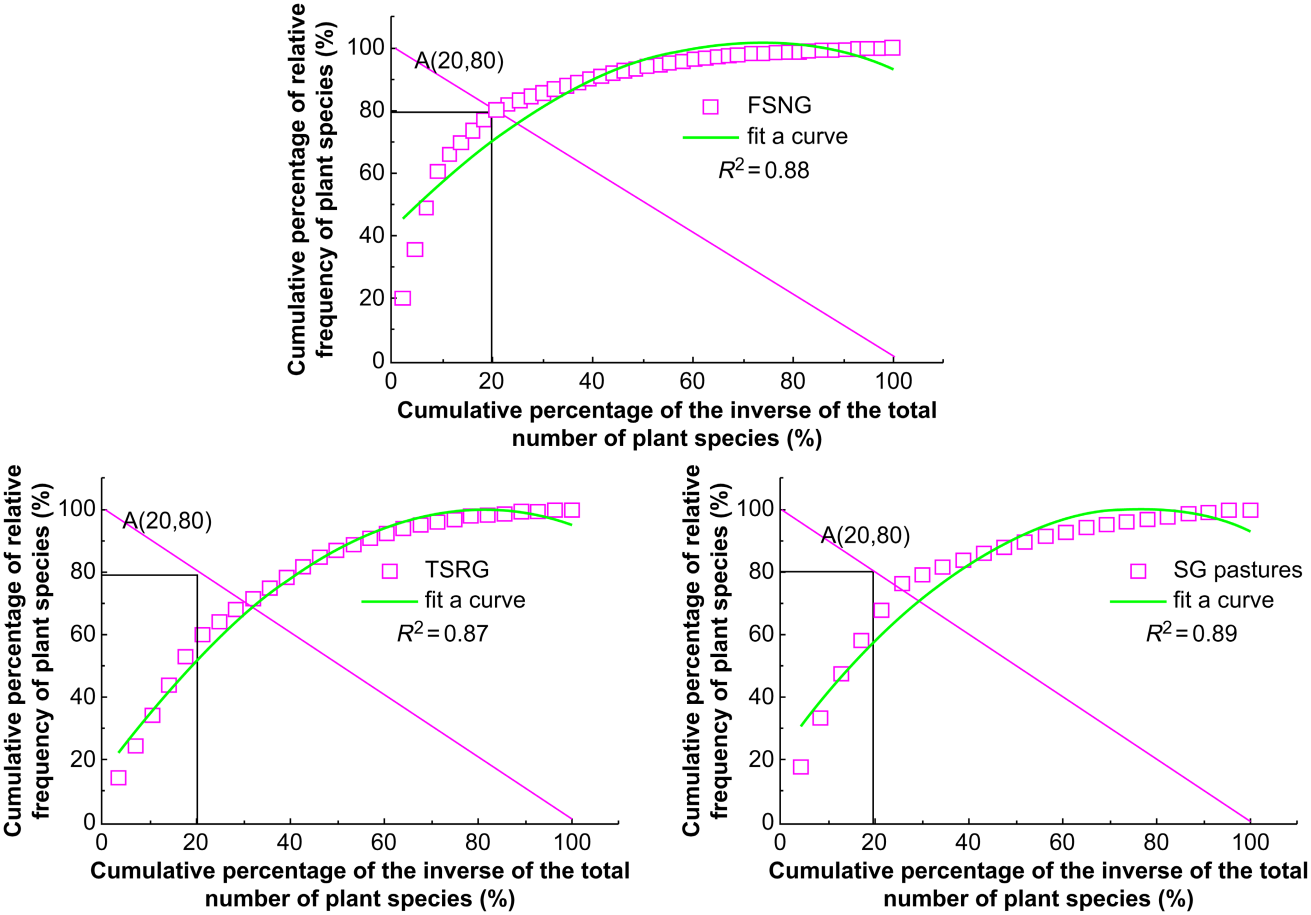
Godron stability index plots for typical steppe plant communities in Inner Mongolia and Mongolia. FSNG, four‐season nomadic grazing; SG, settled grazing; TSRG, two‐season rotational grazing.

The Godron plant community stability calculation method was employed to simulate the community using a smoothing curve (Figure 5). Furthermore, the coordinates of the intersection point of the smoothed curve and a straight line were determined. By integrating the principle of stability from Godron, the proximity of these coordinates (*x*, *y*) to the equilibrium point (20, 80) indicates the community's stability level. Conversely, a greater distance from these coordinates to the equilibrium point suggests lower stability. For visual representation, the Euclidean distance between the intersection point coordinates and those of the equilibrium point is utilized. The Godron stability determination results for communities with different grazing methods indicated that the Euclidean distance of Godron stability determination was 7.16 for FSNG, 13.22 for TSRG, and 16.45 for SG (Table 6). According to the principle, the closer to the equilibrium point (20, 80), the smaller the Euclidean distance, and the higher the stability of the community. The FSNG area was closer to the coordinates of the stabilization point, followed by TSRG, and the SG pastures were the farthest from the coordinates of the stabilization point. Overall, vegetation communities in FSNG pastures were more stable.

**TABLE 6:** Stability analysis of typical steppe communities under different grazing treatments.

Test sample	Fitting curve	*R* ^2^	Intersection coordinates	Euclidean distance
FSNG	*y* = −0.011*x* ^2^ + 1.65*x* + 41.89	0.88	(23.35, 73.67)	7.16
TSRG	*y* = −0.011 ± 1.647 + 41.89	0.87	(29.38, 70.68)	13.22
SG	*y* = −0.013*x* ^2^ + 1.91*x* + 35.00	0.89	(30.77, 67.56)	16.45

Abbreviations: FSNG, four‐season nomadic grazing; SG, settled grazing; TSRG, two‐season rotational grazing.

## 4 Discussion

### 4.1 Different Grazing Patterns and Plant Community Structures

Community structure is an important indicator of the health of grassland plant communities, particularly changes in the dominant species that play a significant role in the direction of grassland ecosystem succession (Chang et al. 2020). First, different grazing practices have a discernible impact on grassland community composition and structural characteristics. In this study, the FSNG grassland had the highest number of species, followed by TSRG and SG had the lowest number of species. This indicates that FSNG has a high species composition diversity and is significantly different from the TSRG and SG communities. Higher grazing rates and consistently higher levels of disturbance resulted in rapid degradation of the pasture, whereas FSNG provided time for pasture restoration and community vegetation recovery due to the degree of mobility of the livestock (Bao, Yi, et al. 2013). Second, in the FSNG area, perennial dominant species, such as *L. chinensis*, *S. grandis*, and *C. duriuscula*, had higher importance values, whereas annuals species had a lower proportion and importance; in addition, the dominance of inchworm moss among the perennial miscellaneous grasses decreased (Wang et al. 2017). In contrast, under SG conditions, the importance value of annual and biennial plants and shrubs and semishrubs increased while the importance value of inchworm moss among the perennial forbs was relatively high. This finding indicated that the plant community is undergoing degradation and succession as a result of the reduction of branches and leaves of the more palatable perennial grasses and the reduction of the effective photosynthetic area due to overfeeding by livestock, resulting in the replacement of perennial grasses by perennial forbs, annual and biennial plants, and shrubs. Overall, FSNG reduces the intensity of livestock disturbance, improves soil physicochemical properties, and promotes vegetation growth (Bao et al. 2014), while SG degrades grassland ecosystems due to long‐term livestock disturbance, which can be restored through the implementation of grazing methods, such as nomadic grazing.

### 4.2 Different Grazing Patterns and Plant Community Characteristics

Differences in grazing patterns affect the degree of grassland vegetation utilization, causing changes in the quantitative features of plant communities (Bat‐Oyun et al. 2016). The density, height, and cover of community vegetation, as the most basic indicators for grassland monitoring are the most intuitive parameters for describing the basic conditions of grasslands as well as auxiliary evaluation indicators in grassland community studies (Schönbach et al. 2011).

In this study, both community cover and community height were higher in the FSNG pasture than the TSRG and SG pastures, suggesting that moderate grazing disturbance and sufficient recreational time positively influence the increase in vegetation cover while rainfall mainly affects vegetation height (Sun, Zhu, and Zhang 2014). However, plant communities under SG conditions exhibited the highest density due to overgrazing, which led to a notable decline in perennial grasses, which are preferred by livestock, and a simultaneous increase in the number of annual and biennial plants, which are less favored by livestock. Additionally, significant differences were observed in the total aboveground biomass of plant communities across different grazing practices, with FSNG pastures displaying notably higher biomass compared to SG pastures. This disparity in biomass could be attributed to grazing pressure reducing plant abundance and subsequently diminishing the overall photosynthetic area, thereby impacting the efficiency of plant organic matter accumulation. Overall, the production performance of FSNG and TSRG was higher than that of SG, indicating that grassland degradation was more pronounced under SG.

### 4.3 Different Grazing Practices and Plant Community Species Diversity

The species diversity of plant communities is a measurable indicator of community structure and closely related to ecosystem functioning. Community diversity changes because of different grazing practices (McIntyre, Heard, and Martin 2003; Grenke et al. 2022). This study demonstrated that the FSNG pastures have the highest values for the three alpha diversity indices, except for the Margalef index, indicating that the communities contained the most information, had the highest number of vegetation species, and were more evenly distributed. Moreover, the FSNG communities were more complex and stable compared with the TSRG and sedentary grazing. The population differences between TSRG and SG were similar, which might be related to the differences in the zonal habitats of the sample sites, the experimental sampling, and their community features. Additionally, the FSNG pastures have lower grazing pressure in summer and are less disturbed, and some of the less palatable plants may affect livestock foraging at a certain stage of growth, which increases their dominance. However, their effects on the community require investigation in fixed‐point continuous experiments.

### 4.4 Current Status of Interspecific Connectivity and Stability of Plants Under Different Grazing Patterns

The overall correlation of plant communities with different grazing practices is a general pattern of interspecific relationships that can reflect the stability and direction of succession of plant communities under different grazing practices (Jian et al. 2018). The stronger the overall association strength, the more robust the mutualism among typical steppe plant species, leading to a more stable community (Qi et al. 2010). Conversely, heightened interspecific competition tends to unfavorably impact plant survival. Upon analyzing the overall interspecific correlation of plant communities, we observed that FSNG pasture communities displayed a positive correlation overall, although it was not statistically significant. This finding indicates that FSNG interspecific associations are loose and somewhat independent and in the stage of continuous improvement; moreover, the plant community structure is still unstable (Jian et al. 2018). Additionally, the Godron plant community stability determination method revealed that FSNG was closer to the coordinates of the stabilization point, followed by TSRG, whereas SG was the farthest away. This disparity arises from the high‐intensity, sustained utilization of grasslands in SG. Overgrazing by domestic animals during the pasture growing season not only curtails the time for compensatory growth of pasture grasses but also prompts further harvesting of deadfalls at the end of the season. Consequently, this leads to a significant reduction in grassland vegetation cover and biomass, resulting in an unstable structure of the plant community. TSRG pastures end grazing earlier in the year compared with SG pastures, thereby allowing more time for compensatory growth after grazing ends and increasing the biomass of pregrazed grasslands more quickly.

FSNG contributes to the stability of the community based on the Godron analysis. SG leads to the disappearance of community species, and species disappearance tends to lead to the loss of a certain plant function from the community. Furthermore, invasive species change the original composition of the community, which has an impact on the stability of the community (Lei et al. 2023).

Our study conclusions are summarized as follows:

1. Grazing mode affects the composition and function of plant communities, and there are differences in the dominant species under different grazing modes. The number of species was higher under FSNG, in which perennial dominant species such as *L. chinensis*, *S. grandis*, and *C. duriuscula* had larger importance values and smaller percentages of annuals and biennials. The number of species in the TSRG and SG pastures was lower, and the perennial forbs *C. squarrosa* and the typical steppe degradation indicator plants *C. duriuscula* and *C. glaucum* appeared in the SG pastures at the same time. Perennials dominated under FSNG, while annuals and biennials were more pronounced under SG pastures.
2. Community cover, community height, and aboveground biomass were higher in FSNG pastures than in TSRG and SG pastures; community densities were the highest in TSRG pastures but not significantly different.
3. Diversity characterization revealed that the Shannon–Wiener, Simpson, and Pielou indexes were higher in the FSNG pastures compared with TSRG and SG pastures, while the Margalef index was highest for TSRG. However, none of these indices showed significant differences. FSNG appeared to support the maintenance of diversity and stability in typical steppe communities, with no significant difference observed between the effects of TSRG and SG on community diversity. Additionally, FSNG, TSRG, and SG plant communities exhibited considerable dissimilarity, showcasing a wide range of structural differentiation and a high degree of habitat variability among the communities.
4. Analysis of interspecific correlation revealed that the FSNG pasture plant communities were positively correlated, with loose interspecific linkages and a certain degree of independence, and in the stage of continuous improvement. The TSRG and SG pastures were negatively correlated and were in the unstable stage of dynamic succession. Stability analysis was used to further explain that FSNG is more stable than TSRG and SG.

## Author Contributions

**Suriguga Gao:** conceptualization (equal), data curation (equal), formal analysis (equal), investigation (equal), writing – original draft (equal). **Yu Hong:** funding acquisition (equal), project administration (equal), resources (equal). **Wulan Tuya:** conceptualization (equal), project administration (equal), writing – review and editing (equal). **Weiqing Zhang:** conceptualization (equal), project administration (equal), supervision (equal). **Chang An:** methodology (equal), project administration (equal), resources (equal). **Siqin Chaoketu:** conceptualization (equal), software (equal), validation (equal). **Xu Sha:** data curation (equal), resources (equal). **Bu He:** data curation (equal), software (equal). **Wu Yinga:** data curation (equal), resources (equal).

## Conflicts of Interest

The authors declare no conflicts of interest.

## Data Availability

The data that support the findings of this study are available from the corresponding author (Wulan Tuya) upon reasonable request.
